# Fruit bagging reduces the postharvest decay and alters the diversity of fruit surface fungal community in ‘Yali’ pear

**DOI:** 10.1186/s12866-022-02653-4

**Published:** 2022-10-05

**Authors:** Congcong Gao, Yang Zhang, Huimin Li, Qi Gao, Yudou Cheng, Solabomi Olaitan Ogunyemi, Junfeng Guan

**Affiliations:** 1 Institute of Biotechnology and Food Science, Hebei Academy of Agriculture and Forestry Sciences, Shijiazhuang, 050051 China; 2 Plant Genetic Engineering Center of Hebei Province, Shijiazhuang, 050051 China; 3 State Key Laboratory of Rice Biology, Ministry of Agriculture Key Lab of Molecular Biology of Crop Pathogens and Insects, Institute of Biotechnology, Zhejiang University, Hangzhou, 310013 China

**Keywords:** Postharvest decay, Fruit bagging, Fungal community, ‘Yali’ pear, *Pichia*

## Abstract

**Background:**

Fruit bagging is an effective technique for fruit protection in the orchard management. Bagging can create a micro-environment for fruit growth and affect fruit quality during storage, in which the diversity of microorganisms may play an important role. Therefore, various methods including biochemistry, analytical chemistry, and bioinformatics methods were used to reveal the influences of fruit bagging on postharvest fruit quality, physiological characters, decay and surface fungal community of ‘Yali’ pear fruit were investigated in this study.

**Results:**

Fruit bagging significantly decreased the postharvest decay after 15 days of ambient storage. There were no significant differences in fruit firmness, titratable acid and ethylene production rate between the fruit-bagging and non-bagging group after 15 days of storage, while the soluble solids contents (SSC) and respiration rate in non-bagging fruit was significantly higher than that in fruit-bagging after 15 days of storage. Furthermore, the surface microbes of pear were collected and determined by the new generation sequencing technology. The alpha diversity of fungi in non-bagging fruit decreased significantly after 15 days of storage, while there were no significant changes in bagging fruit. *Ascomycota* and *Basidiomycota* were the two major phyla detected in the bagging fruit, and the dominant fungal genera were *Alternaria* (23.7%), *Mycosphaerella* (17.25%), *Vishniacozyma* (16.14%), and *Aureobasidium* (10.51%) after 15 days of storage. For the non-bagging pear, *Ascomycota* was the only phylum detected, and the dominant genera was *Pichia* (83.32%) after 15 days of storage. The abundance of *Pichia* may be regarded as the biomarker to indicate the degree of fruit decay.

**Conclusions:**

This study showed that fruit bagging could significantly reduce postharvest fruit decay and respiration rate of ‘Yali’ pear. Significant differences were found in fungal composition between bagging and non-bagging pear after storage for 0 or 15 days. Fruit bagging maintained the diversity of fungi on the fruit surface, increased the abundance of non-pathogenic fungi, and even antagonistic fungi such as *Aureobasidium*, *Vishniacozyma*, and *Mycosphaerella*. A reduction in the abundance of pathogenic fungi and incidence of postharvest decay during the storage of ‘Yali’ pear were also recorded. In conclusion, fruit-bagging changed the fungal diversity on fruit surface of ‘Yali’ pear, which had significant effect on reducing postharvest fruit decay, and thus prolong the storage period of ‘Yali’ pears. The future thrust of this study will focus on the isolation of fungi or bacteria from pear fruit surface and identify their roles in causing fruit decay and changing fruit quality during storage.

## Introduction

Pears are favored by consumers for its richness in dietary fiber, mineral elements, ascorbic acid and other nutrients [1]. However, postharvest storage of pear fruits faces many challenges, among which fruit decay caused by pathogenic fungi often causes significant economic losses.

Most of the pathogenic fungi identified during storage are infected in orchards, showing latent infection, and finally broke out in the storage period. *Penicillium expansum*, *Botrytis cinerea*, *Mucor piriformis*, *Phialophora malorum*, *Alternaria* spp., *Cladosporium herbarum* and *Neofabrea* spp. are common pathogenic fungi found in pears [2–5]. During postharvest storage, pathogens may invade and cause postharvest fruit decay when conditions are suitable [6]. Some pathogenic fungi such as *Alternaria, Aspergillus, Penicillium*, and *Fusarium* may also produce mycotoxins, which cause harm to human and animal health and bring huge safety risks to consumers [7, 8]. Therefore, it is very important to use effective prevention measures to minimize fruit decay during storage.

Fruit bagging, a safe and environmentally friendly technology, is used by many countries, which can effectively reduce pesticide residues, resist the harm of diseases and insects and improve fruit color [9]. The ‘Yali’ pear (*Pyrus bretschneideri* Rehd. cv. Yali) is a famous cultivar in China [10]. However, the effects of fruit bagging on surface microbiota of ‘Yali’ pear and its role in postharvest fruit decay remain unclear. Studies have shown that the interaction between antagonists, pathogens, and host affects the occurrence of disease [11, 12], prompting us to study the relationship between fruit bagging and postharvest diseases with a new perspective. The purposes of this study were to investigate the effects of fruit bagging in field on postharvest decay and fungal community of fruit surface, and to further analyze the control mechanism of diseases during storage in ‘Yali’ pear.

## Materials and methods

### Fruit bagging and collection

Field experiments were conducted in Zhaoxian orchard Shijiazhuang, China (115.018171°E, 37.781454°N). The ‘Yali’ pear (*Pyrus bretschneideri* Rehd. cv. Yali) trees which were 50 years old were used. The double-layer paper bags which were black outside and white inside were used for fruit bagging on May 23, 2021, for the forty-five days after bloom, and the non-bagging as control. No pesticide was applied before bagging, while abamectin was applied after bagging to protect the fruit from *Psylla chinensis*. The fruit were harvested on September 16, 2021, using sterile PE gloves and they were placed in sterile fresh-kept bags (produced by the Institute of Agricultural Products and Fresh-keeping of Shanxi Academy of Agricultural Sciences with good CO_2_ permeability). They were afterward transported to the laboratory within 2 hours for subsequent storage at ambient temperature (25 ± 2 °C).

Fruit were stored in sterile fresh-kept bags at 25 ± 2 °C with a humidity of 95% (humidity recorder RC-4HC, Jingchuang, China). Fungal samples on fruit surface were collected by wiping with cotton swabs after 0 and 15 days of storage [13]. Bagging groups stored for 0 and 15 days were set as B0 and B15, respectively, while non-bagging group stored for 0 and 15 days were set as NB0 and NB15, respectively.

### Determination of decay index

The decay was divided into four grades. Grade 0: the fruit is intact without decay; Grade 1: decayed area was less than 25% of the total fruit surface area; Grade 2: decayed area was between 25 to 50% of the total fruit area; Grade 3: decayed area was more than 50% of the total fruit area. Decay index was calculated according to the following formula: decay index = ∑ (number of decay fruit at each level × grade value of corresponding level) / (total fruit number × highest grade value). Each treatment had five replicates with 10 fruit per replicate.

### Assessment of fruit quality

For firmness (N) determination, 15 fruit were randomly selected and peeled at the equatorial part of the fruit. Both sides of each fruit were determined by GY-4 Firmness Meter (Tuopu, China). Soluble solids content (%) was measured using a PAL-1 handheld digital saccharimeter (ATGAO, Japan). Titratable acidity (%) was determined by the method of acid-base titration. Each experiment contained 5 replicates.

### Determination of respiration and ethylene production rates

For respiration rate (mg h^− 1^ kg^− 1^), pear fruit were sealed in a closed container for 30 min, then 10 ml of mixed gas was extracted and CO_2_ analyzed using an infrared CO_2_ analyzer (HWF-1A, Kexi Instruments, China). For ethylene production rate (μL kg^− 1^ h^− 1^), 1 mL of mixed gas was extracted after the pear fruit were sealed for 5 h, then injected into Gas Chromatograph (GC-9790II, Fuli Instruments, China) to determine the concentration of ethylene. Each treatment had three replicates.

### ITS1 sequencing and bioinformatics analysis

The ITS1 region of rRNA gene was amplified using the universal fungal primers ITS1-F (5′-CTT GGT CAT TTA GAG GAA GTA A-3′) / ITS1-R (5′-GCT GCG TTC TTC ATC GAT GC-3′) [14]. The high-throughput sequencing of PCR products was performed on an Illumina MiSeq/NovaSeq platform at Personal Biotechnology, Shanghai, China.

Alpha diversity was calculated as implemented in the QIIME2 to analyze the complexity of species diversity [15]. The diversity indices including Chao1, Good’s nonparametric coverage, Shannon indices, Pielou’s evenness and Observed species were calculated based on observed ASVs among all groups. Principal coordinates analysis was used to demonstrate beta diversity using the Bray-Curtis distance algorithm, and the PERMANOVA (adonis) test for differences were analysed by using ImageGP [16]. After, all samples were adjusted to the same sequencing depth, the fungal composition of all treatments at each taxonomic level was done using taxa barplot function of QIIME2. LEfSe (Linear Discriminant Analysis Effect Size) was employed to find different species between groups, which are commonly referred to as biomarkers, using the online workflow of genescloud (https://www.genescloud.cn). One-against-all comparison strategy was used and the threshold of linear discriminant analysis was set as 4. The canonical correspondence analysis (CCA) was conducted by the genescloud tools.

### Statistical analysis

The graphs including fruit decay index, quality, and physiological characteristics were generated using the GraphPad Prism 9 software (GraphPad Inc., CA, United States). The significant differences between treatments were tested by two-way analysis of variance (ANOVA), and differences were considered to be significant at *P* < 0.05 (*), *P* < 0.01 (**), and *P* < 0.001 (***).

## Results

### Fruit bagging reduces postharvest decay

**Fig. 1:**
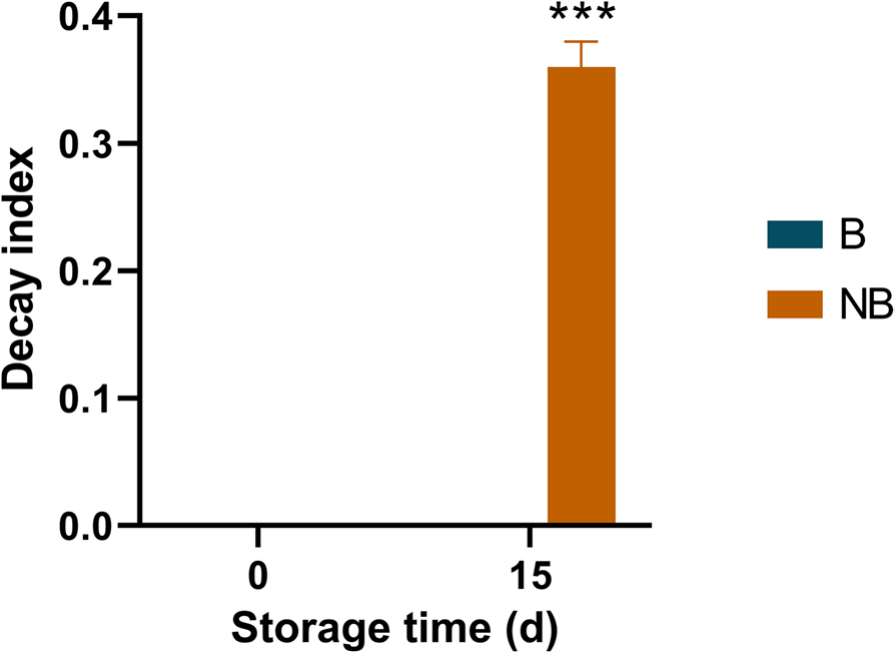
The effect of fruit bagging on postharvest decay index in ‘Yali’ pear. B: bagging; NB: non-bagging

The results showed that fruit bagging significantly reduced postharvest decay (Fig. 1). Specifically, bagging fruit remained healthy while the decay index of non-bagging fruit reached 0.36 after 15 days of postharvest storage, which was significantly higher than that of bagging fruit.

### Fruit bagging affects postharvest fruit quality

**Fig. 2:**
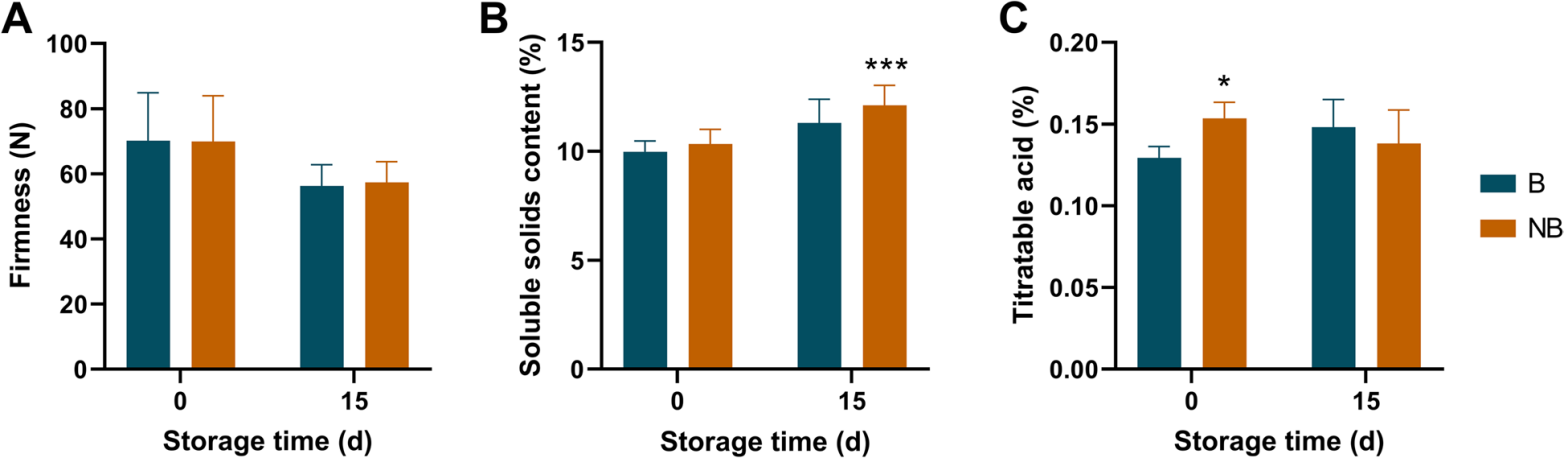
Effects of fruit bagging on firmness (**A**), soluble solids content (**B**) and titratable acid content (**C**) of ‘Yali’ pear. B: bagging; NB: non-bagging

The effect of fruit bagging on postharvest quality was further determined. Fruit firmness decreased significantly after 15 days of storage, but there was no significant difference in bagging fruit compared with control (non-bagging fruit) (Fig. 2A). For SSC, there was no significant difference between bagging and non-bagging fruit at the beginning of storage (Fig. 2B). However, the SSC of non-bagging fruit (12.11%) was significantly higher than that of bagging fruit (11.3%) after 15 days of storage. Titratable acid content of non-bagging fruit (0.15%) was significantly higher than that of bagging fruit (0.13%) at the beginning of storage, but there was no significant difference after 15 days of storage (Fig. 2C).

### Fruit bagging affects physiological characteristics of pear fruit

**Fig. 3:**
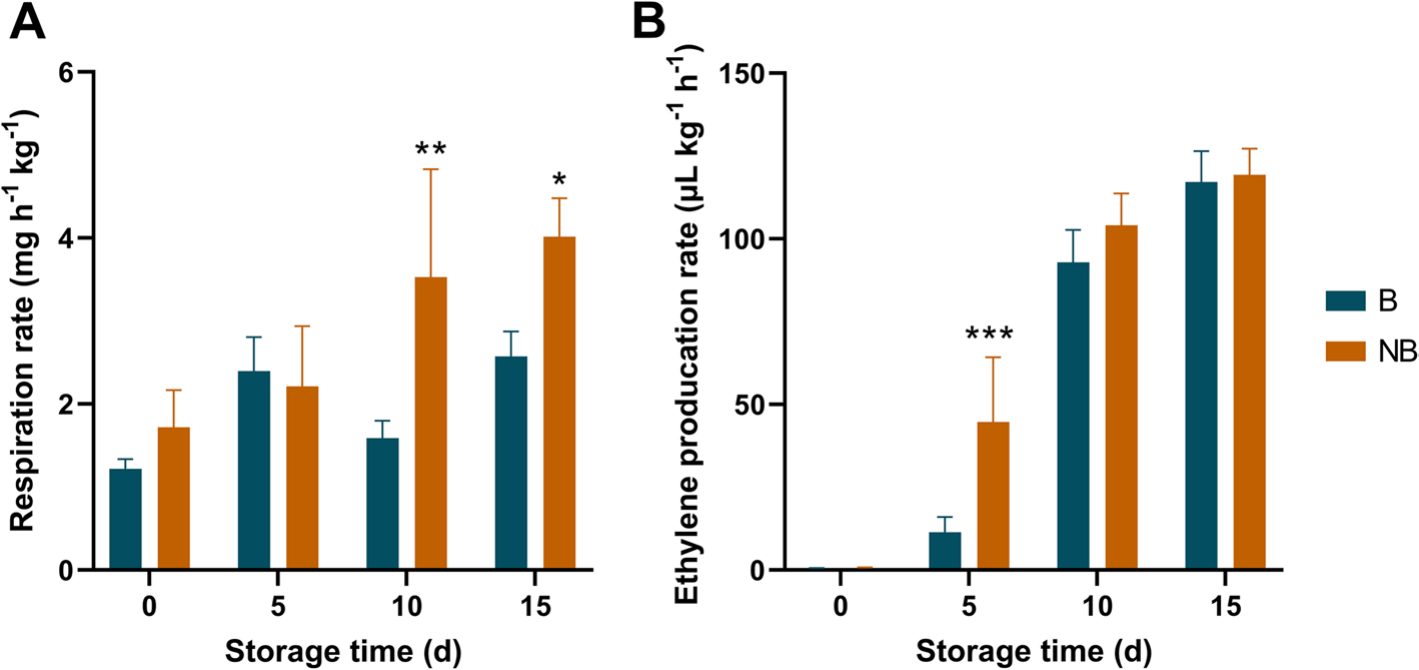
Effects of fruit bagging on respiration rate (**A**) and ethylene production rate (**B**) of ‘Yali’ pear during storage at ambient temperature. B: bagging; NB: non-bagging

Fruit respiration rate and ethylene production rate were used as two indicators of fruit physiological characteristics. In order to accurately investigate the effect of field bagging on postharvest physiological characteristics of fruit, we determined the respiration rate and ethylene production rate of fruit every 5 days. The results showed that there was no significant difference in respiration rate between bagging and non-bagging fruit before 5 days of storage, while there was a significant difference between the two groups after 10 days of storage, and the respiration rate of non-bagging fruit was significantly higher than that of bagging fruit (Fig. 3A). Further results showed that the ethylene production rate of non-bagging fruit was significantly higher than that of bagging fruit after 5 days of storage, and reached the level of bagging fruit after 10 days of storage (Fig. 3B).

### Fruit bagging changed fungal diversity on the surface of pear fruit

#### Alpha diversity analysis

**Fig. 4:**
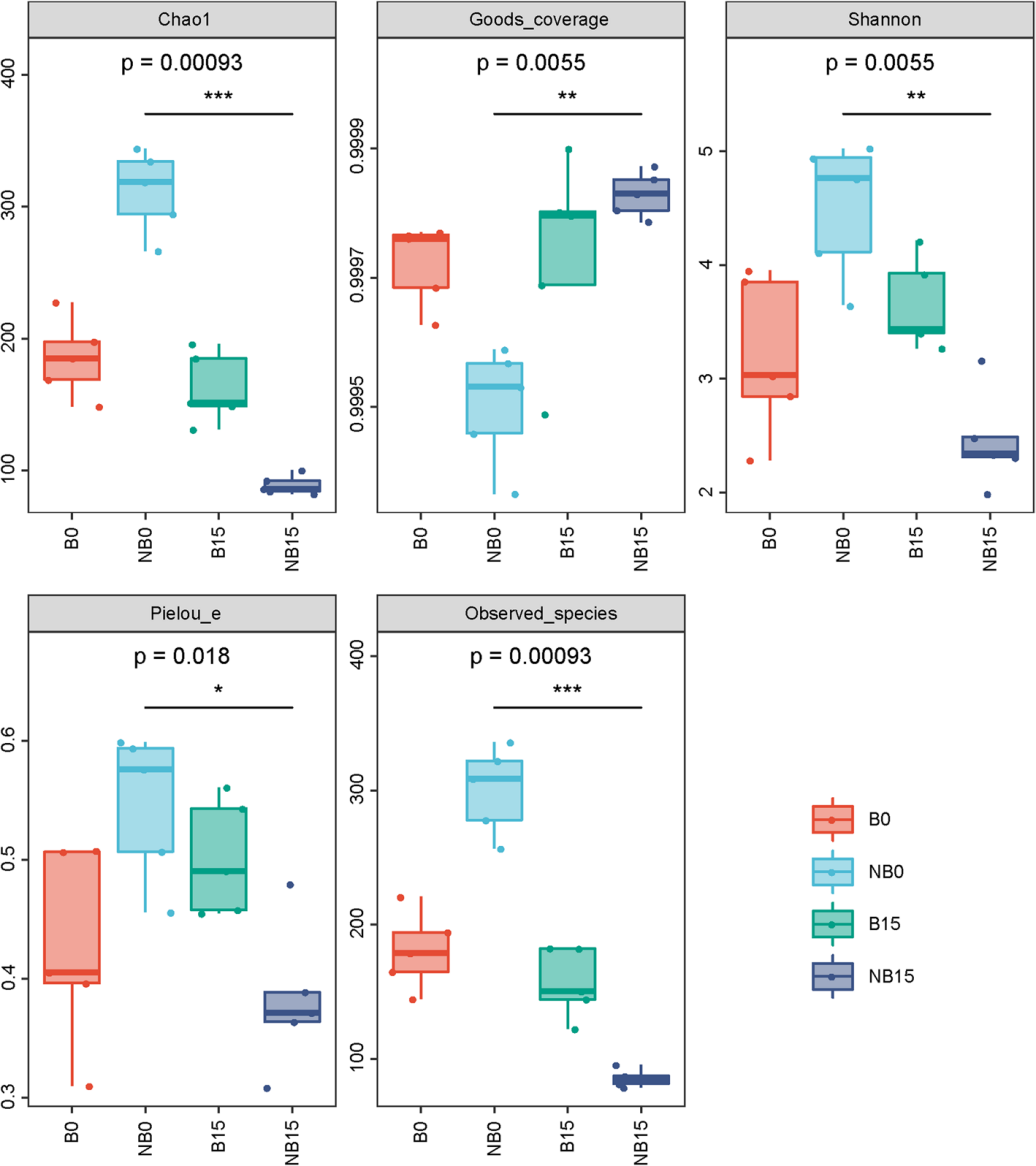
Alpha diversity of fungal community on surface of ‘Yali’ pear. Chao1 and Observed species index indicate the richness of fungal community; Shannon index represents the fungal diversity; Pielou was used to characterize evenness of fungal community; Good’s Coverage indicates the amount of determined species. B: bagging; NB: non-bagging

Five indexes, including Chao1, Good’s coverage, Shannon, Pielou, and Observed species, were used to show the effect of bagging on the alpha diversity of fungal community on fruit surface. The results showed that the diversity and richness of fungal species on the surface of non-bagging fruit were significantly higher than that on the surface of bagging fruit at harvest (Fig. 4). The surface fungal diversity of non-bagging fruit decreased significantly, while there was no significant change in bagging fruit after storage for 15 days.

#### Beta diversity analysis

**Fig. 5:**
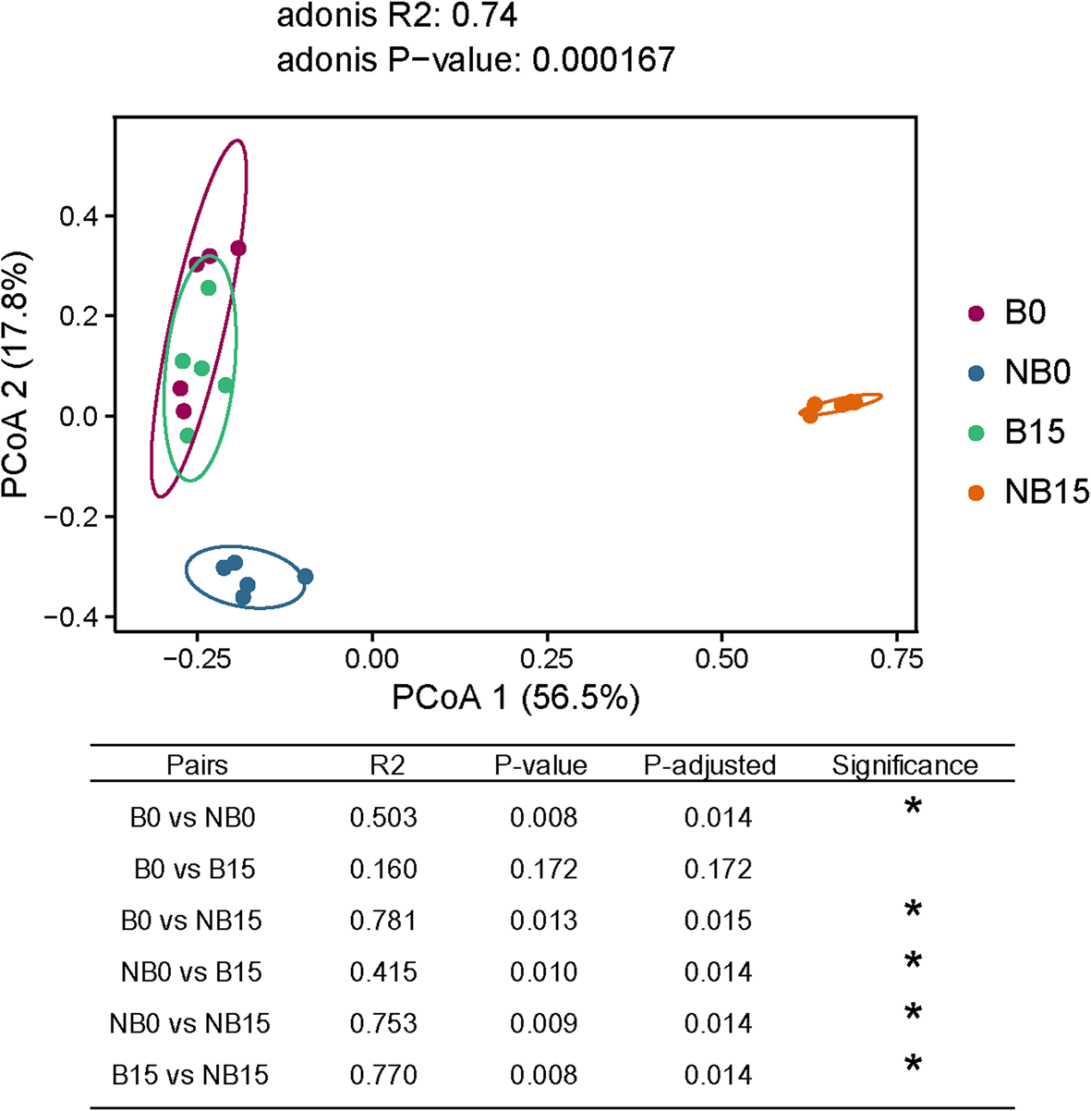
Beta diversity of fungal community on surface of bagging or non-bagging ‘Yali’ pear with 0 or 15 days of storage. B: bagging; NB: non-bagging. The PERMANOVA (adonis) test was used for identifying the differences

According to the results of beta diversity, the bagging (B0) and non-bagging groups (NB0) at the beginning of storage could be clearly separated on PCoA2, indicating that fruit bagging significantly affected the fungal community on the surface of pear at harvest (Fig. 5). After 15 days of storage, fruit bagging (B15) and non-bagging (NB15) group could also be clearly differentiated on PCoA1, indicating that fruit bagging significantly affected the fungal community on the surface after storage. However, bagging fruit after 15 days of storage (B15) could not be clearly differentiated in PCoA1 or PCoA2 from 0 day of storage (B0), while non-bagging fruit after 15 days of storage (NB15) could be clearly differentiated in PCoA1 from 0 day of storage (NB0). This result indicates that bagging treatment could maintain the fungal diversity on fruit surface of ‘Yali’ pear.

### Fungal composition on the surface of pear fruit

**Fig. 6:**
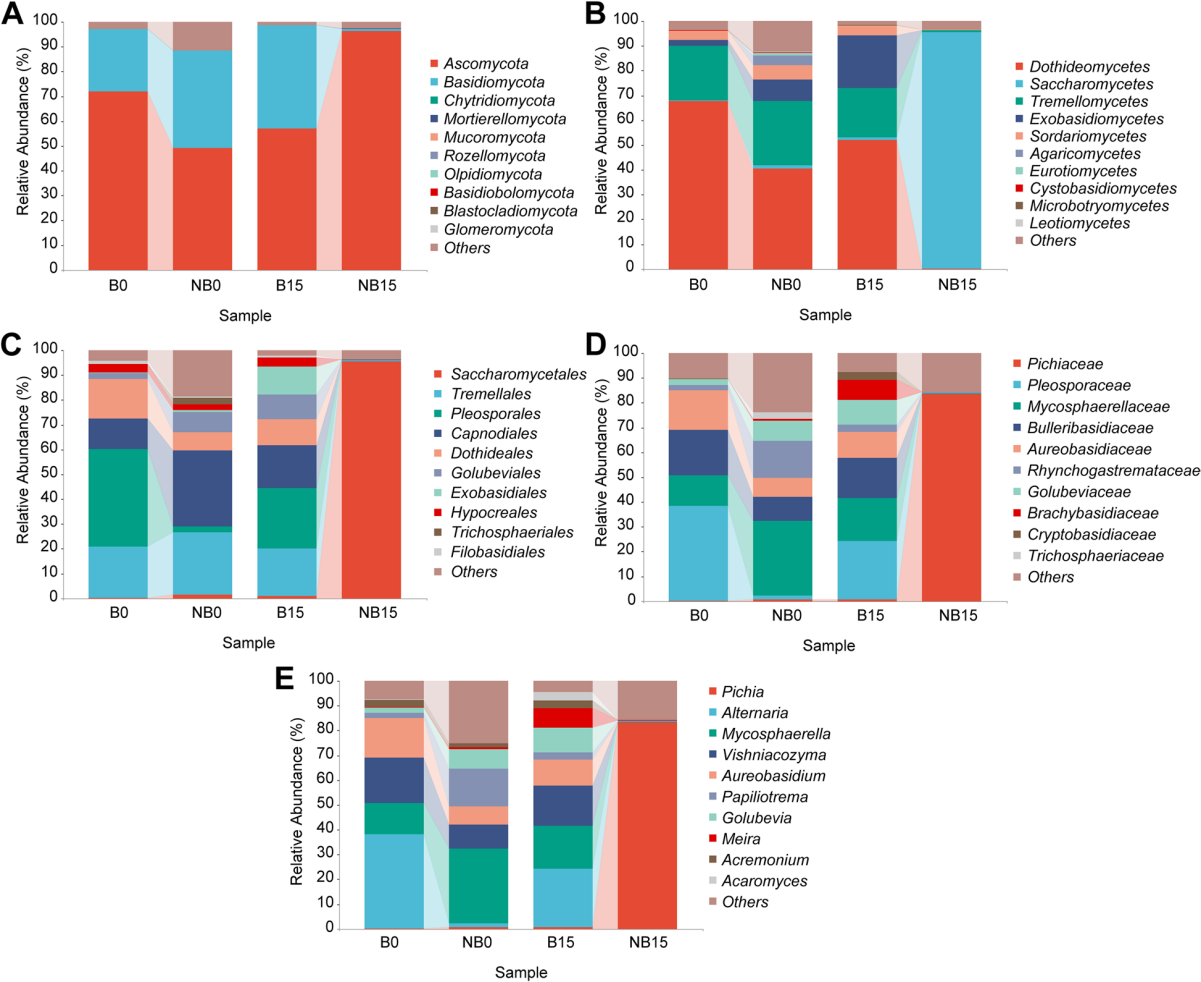
Fungal composition on surface of ‘Yali’ pear at phyla (**A**), class (**B**), order (**C**), family (**D**) and genus (**E**) level. B: bagging; NB: non-bagging

The main fungal composition on pear fruit surface at phyla, class, order, family and genus level was shown in Fig. 6. At the initial storage stage of the bagging group, *Ascomycota* (72%) and *Basidiomycota* (25%) were the dominant phyla, and the main classes were *Dothideomycetes* (68%), *Tremellomycetes* (22%), *Sordariomycetes* (3.5%), and *Exobasidiomycetes* (2.5%). The most abundant fungal genera were *Alternaria* (38.2%), *Aureobasidium* (16%), *Vishniacozyma* (18.2%), *Mycosphaerella* (12.5%), *Acremonium* (3%), *Papiliotrema* (2.2%), and *Golubevia* (2.1%). *Ascomycota* (49%), and *Basidiomycota* (39%) were the dominant phyla in the non-bagging group at the initial storage stage. The main classes were *Dothideomycetes* (40%), *Tremellomycetes* (26%), *Sordariomycetes* (5.7%), *Exobasidiomycetes* (8.8%), and *Agaricomycetes* (3.9%). The most abundant genera were *Mycosphaerella* (30.3%), *Papiliotrema* (15%), *Aureobasidium* (7.4%), *Vishniacozyma* (9.6%), *Golubevia* (8%), and *Acremonium* (1.6%).

After 15 days of storage, *Ascomycota* (96%), *Saccharomycetes* (95%), *Saccharomycetates* (95.4%), *Pichiaceae* (83.5%), and *Pichia* (83.3%) were the dominant phyla, class, order, family and genus in the non-bagging group, respectively. On the other hand, the fungal composition on the surface of bagging fruit was much more diverse. *Ascomycota* (57%), and *Basidiomycota* (42%) were the dominant phyla. *Dothideomycetes* (52%), *Tremellomycetes* (20%), *Exobasidiomycetes* (21%), and *Sordariomycetes* (3.8%) were the main classes. *Hypocreales* (3.7%), *Exobasidiales* (11.4%), *Golubeviales* (9.8%), *Dothideates* (10.5%), *Capnodiales* (17.3%), *Pleosporales* (24.3%), and *Tremellales* (19.3%) were the dominant orders. *Cryptobasidiaceae* (3.3%), *Golubeviaceae* (9.8%), *Rhynchogastremataceae* (3%), *Aureobasidiaceae* (10.5%), *Bulleribasidiaceae* (16%), *Mycosphaerellaceae* (17.3%), and *Pleosporaceae* (23.7%) were the dominant families. *Acaromyces* (3.3%), *Acremonium* (3%), *Meira* (8%), *Golubevia* (9.8%), *Papiliotrema* (3%), *Aureobasidium* (10.5%), *Vishniacozyma* (16%), *Mycosphaerella* (17.2%), and *Alternaria* (23.7%) were the main genera.

### Biomarker identification by LEfSe

**Fig. 7:**
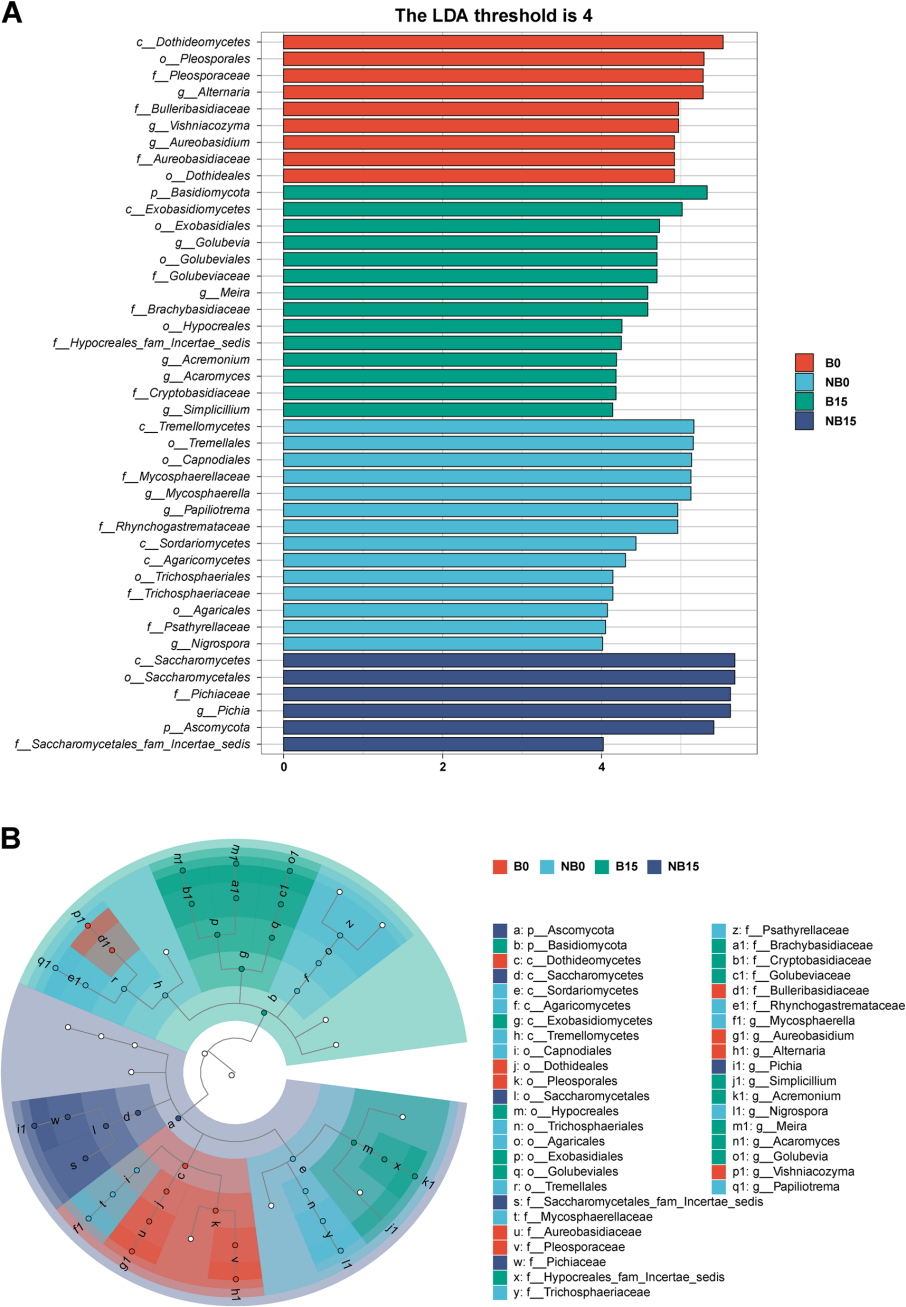
Fungal biomarkers on the surface of pear of different groups by linear discriminant analysis effect size (LEfSe). A: Histogram of LDA value distribution. B: Taxonomic cladogram. The threshold of linear discriminant analysis was set as 4. B: bagging; NB: non-bagging

Linear discriminant analysis effect size (LEfSe) was used to analyze the fungal biomarkers on the surface of pear different groups. As shown in Fig. 7, biomarkers in all groups at different taxonomic levels were identified. *Alernaria*, *Vishniacozyma*, and *Aureobasidium* can be regarded as the biomarkers of B0 group. There are also three fungal genera that can be used as biomarkers in the NB0 group: *Mycosphaerella*, *Papiliotrema*, and *Nigrospora*. Five fungal genera including *Golubvia*, *Meira*, *Acremonium*, *Acaromyces*, and *Simplicillium* were identified as biomarkers for the B15 group. The biomarker of NB15 group was *Pichia*. Since the NB15 group had the highest degree of fruit decay, *Pichia* had the potential as a biomarker of fruit decay.

### Differences in the abundance of the yeast

**Fig. 8:**
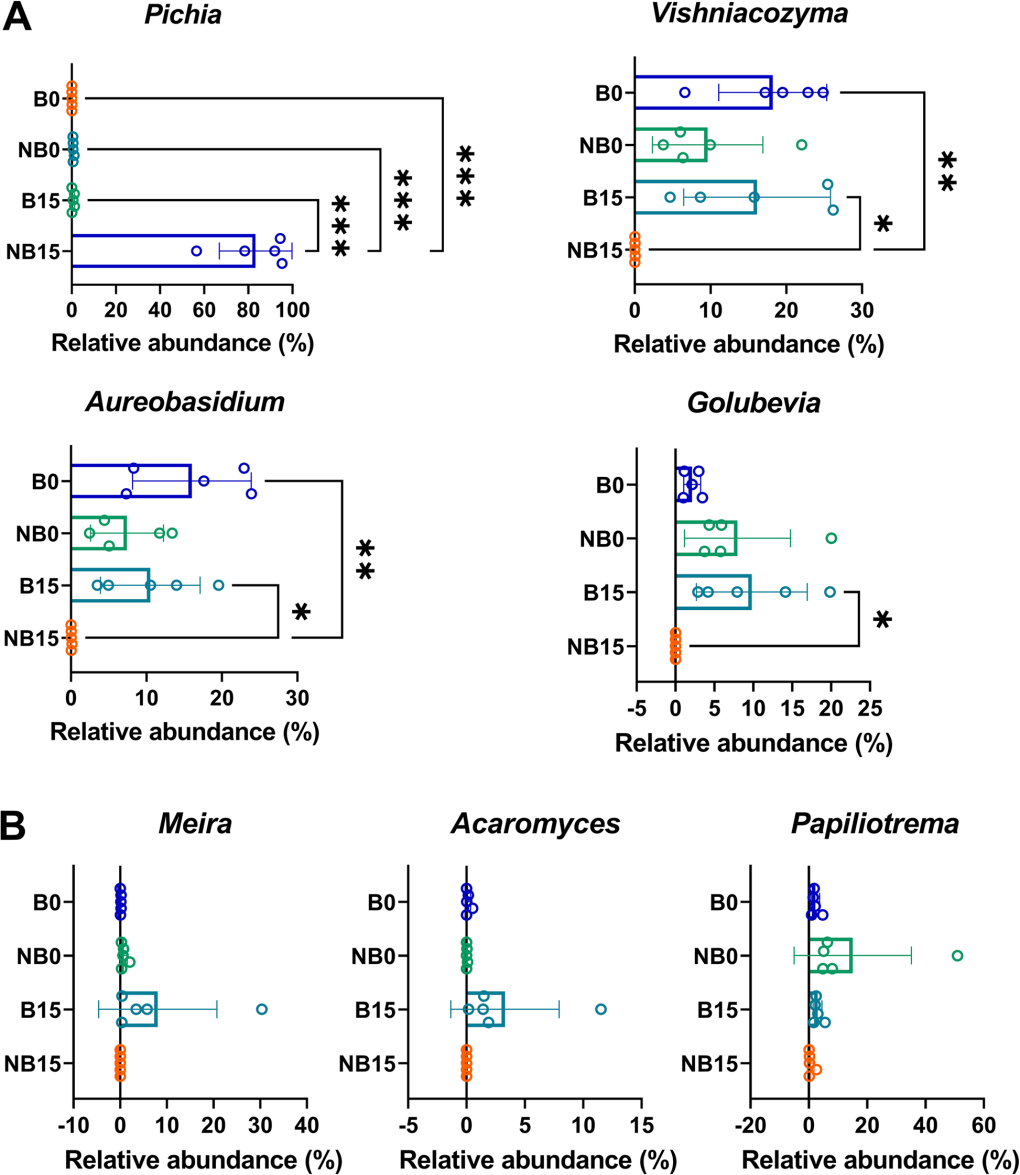
Differences in the abundance of yeasts between bagging and non-bagging groups in ‘Yali’ pear

Because the functions of yeast are complex and many of them act as healthy fungi antagonizing pathogens, the differences in the abundance of yeast between different groups were analyzed. As shown in Fig. 8, the relative abundance of *Pichia* was significantly higher in NB15 than other groups, while yeasts including *Vishniacozyma*, *Aureobasidium*, and *Golubevia* showed significant higher abundance in B15 than other groups. Hence, indicating they might play positive roles in healthy fruit. On the other hand, the abundance of fungi including *Meira*, *Acaromyces*, and *Papiliotrema* showed no significant differences between groups.

### Correlation analysis of postharvest fruit decay and microbial diversity

**Fig. 9:**
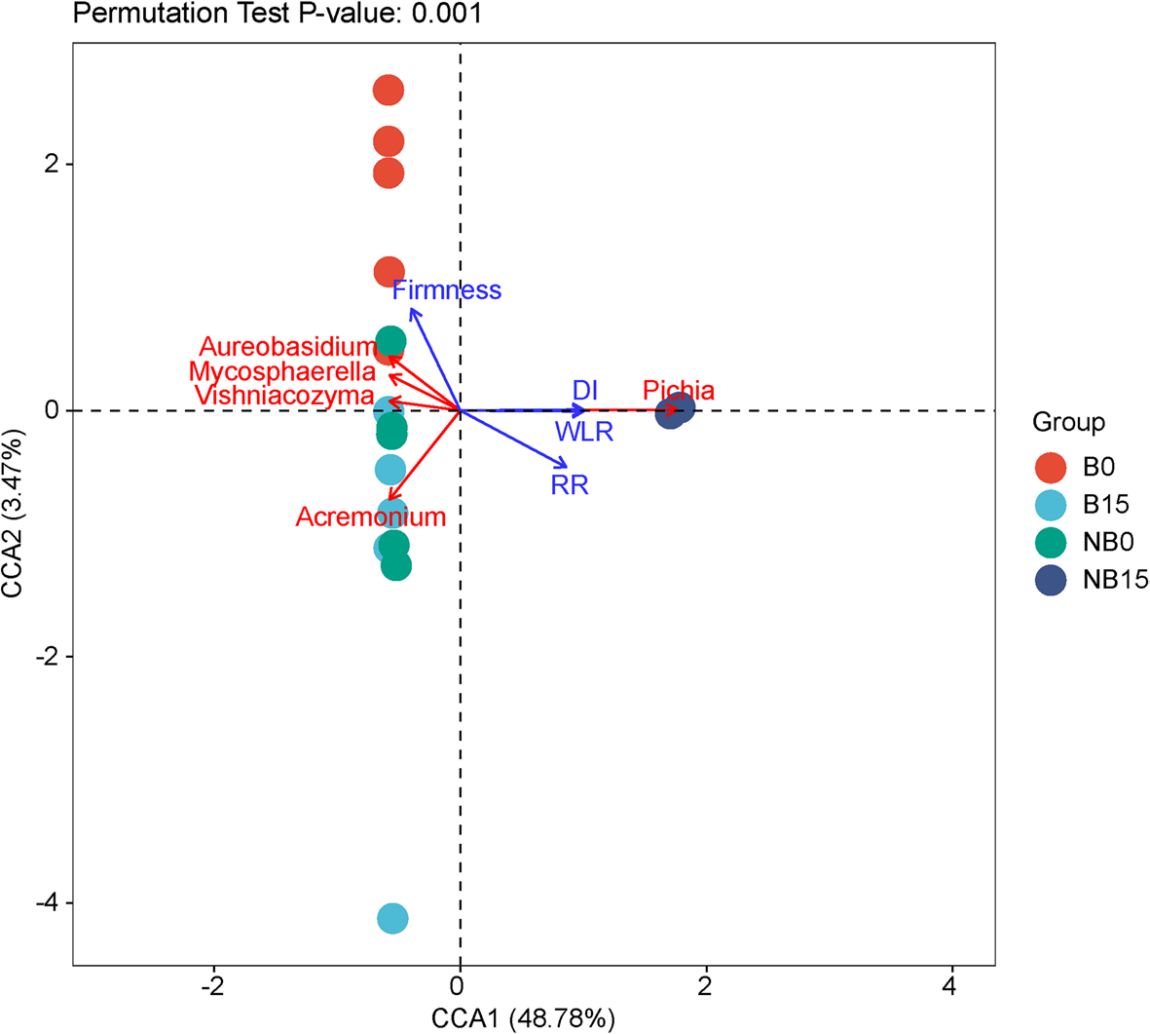
Correlation of postharvest fruit decay and microbial diversity by canonical correspondence analysis in ‘Yali’ pear. DI: Decay index. RR: Respiratory rate. B: bagging; NB: non-bagging

The canonical correspondence analysis (CCA) was performed to show the relationships of bagging, storage time, microbial diversity, and fruit qualities. Given that fruit firmness, respiration rate, and decay index were significantly affected by bagging, they were used as representative indicators of fruit quality in CCA. The results showed that the fungal community of non-bagging fruit stored for 15 days was positively correlated with respiration rate and decay index, while negative correlation was recorded for fruit firmness. This result indicates that bagging and storage time had significant influence on the correlation between fungal community and fruit quality (Fig. 9). Moreover, decay index and respiratory rate were negatively correlated with the abundance of *Aureobasidium*, *Vishniacozyma*, and *Mycosphaerella*, while positively correlated with the abundance of *Pichia*. The results of fruit firmness were the opposite of those of respiration rate and decay index. The abundance of *Acremonium, Aureobasidium*, *Vishniacozyma*, and *Mycosphaerella* shows negative correlations with *Pichia*, suggesting that *Pichia* may have different functions from these fungi during the storage of ‘Yali’ pear fruit.

## Discussions

Fruit bagging has important effects on postharvest fruit storage quality and physiological characteristics [17]. We observed that fruit bagging had no significant effect on firmness of ‘Yali’ pear after 15 days of storage, but significantly affected SSC, titrable acid, and respiration rate of fruit. The increase of respiration rate indicated that the fruit became ripe, and the changes of SSC and titrable acid may be due to the conversion of sugars and acids during fruit ripening. In ‘Red Fuji’ apple, Xia et al. reported that no significant effect of fruit bagging on the content of SSC but reducing sugar and titrable acid [18]. Considering the genetic and physiological differences between apple and pear, they may respond to fruit bagging differently. Therefore, fruit bagging may have different effects on fruit quality in different fruits.

Fungi community richness of bagging fruit is significantly higher than that of non-bagging fruit before storage, which may be due to the little variations in temperature and relative humidity after bagging [19, 20]. The micro-environment inside fruit bag is controlled, hence, microbial competition is relatively harmonious, and the community does not change drastically due to environmental changes. In addition, abamectin was used after fruit bagging. Although no effect of abamectin on phyllospheric fungi was found, it however, significantly affect bacterial diversity [21]. The effect of pesticides on fungal diversity of fruit surface in non-bagging pear should be noticed. Therefore, the decrease of fungal diversity on fruit surface of non-bagging pear was probably resulted by the use of abamectin as well. Most of the pathogens colonize the fruit surface in the form of latent infection and will not cause disease in the short term, thus maintaining the diversity of fungal community on the fruit surface. It was found that bagging significantly reduced postharvest decay after storage for 15 days, and the fungal diversity of bagging group was higher than that of non-bagging group. Thus, indicating that the dominant strains that appeared were associated with fruit decay.

Fungal composition on the surface of pear fruit showed that the main fungi on the surface of pear were *Ascomycota*, which grow fast and can survive harsh conditions with low nutrient level [22]. They can adapt to a wide range of substrates in challenging environments such as ultraviolet light, water and high temperature stress [23]. *Ascomycota* has been shown to be pathogenic fungi in plants and insects [24]. However, *Mycosphaerella* was dominant in non-bagging group at the beginning of the storage period which was significantly different from the bagging group. *Mycosphaerella* is widely distributed on trees, herbaceous plants, and cultivated crops such as saprophytes, plant pathogens, or endophytes [25–28]. It has been reported *Mycosphaerella* can cause leaf diseases such as leaf spot, leaf litter, and branch blight [29–32]. In addition, *Mycosphaerella* sp. has been reported to be the causal organism of pear skin stain which had a stronger pathogenicity than *Penicillium* spp. and *Alternaria* spp. [33]. *Pichia* was found to be the dominant genus in the non-bagging group after storage for 15 days. *Pichia* has been reported to have a negative impact on the growth of *Zygosaccharomyces* spp., *Botrytis cinerea*, and *Brettanomyces bruxellensis*, and it could secrete toxins or organic acids, which were lethal to other yeasts and filamentous fungi [34–37]. These characteristics therefore, gave it an advantage to become the dominant species by negatively affecting the growth of other fungi.

Bagging helped to maintain the fungal diversity on the fruit surface of ‘Yali’ pear after storage for 15 days. In addition to common pathogenic fungi such as *Alternaria*, *Mycosphaerella*, and *Acremonium* [38–41]. A number of yeast or yeast-like fungi including *Vishniacozyma*, *Aureobasidium*, *Papiliotrema*, *Golubevia*, *Meira*, and *Acaromyces*, which have been reported to have a variety of functions were also observed in this study. *Vishniacozyma* was reported to have an inhibitory effect on *Penicillium*, which can control blue mold in apple, and is widely existed [42, 43]. *Aureobasidium* was also reported for its effectiveness against blue mold caused by *Penicillium expansum* in stored apple fruit, and can be found in a variety of environments and with a worldwide distribution from cold to warm climates [44, 45]. *Papiliotrema* was found to be present in plant leaves which was non-pathogenic, producing β-galactosidase [46, 47]. *Golubevia* has been found to regulate the plant defense system and has certain antagonistic effect on powdery mildew of cucumber [48]. *Acaromyces* and *Meira* was reported to be the pathogens causing fruit stain of Japanese pear [49]. Result from this study has shown that the abundance of *Vishniacozyma*, *Aureobasidium*, and *Golubevia*, which have been reported to have antagonistic effects, were significantly higher in B15 than NB15. The abundance of *Vishniacozyma* and *Aureobasidium* were positively correlated with fruit firmness. Therefore, *Vishniacozyma* and *Aureobasidium* were supposed to be regarded as the healthy fungi after fruit bagging.

Previous results have shown that fruit bagging significantly increased fungal diversity and promote healthy fungal communities which protect fruit from the invasion of pathogens [50]. This report [50] is however, consistent with the results of this study. Therefore, fruit bagging has generated a diverse fungal community on the fruit surface of pear, in which antagonistic yeasts were relatively abundant, which may be involved in inhibiting the reproduction of pathogens and reducing the occurrence of postharvest decay.

## Conclusions

This study showed that fruit bagging could significantly reduce postharvest fruit decay and respiration rate of ‘Yali’ pear, and affect other fruit qualities and physiological characteristics. The richness, evenness, and diversity of the fungal community on pear surface were higher in bagging group than in non-bagging one, and significant differences were found in fungal composition between bagging and non-bagging pear after storage for 0 or 15 days. Hence, fruit bagging maintained the diversity of fungi on the fruit surface, increased the abundance of non-pathogenic fungi, including antagonistic fungi such as *Aureobasidium*, *Vishniacozyma*, and *Mycosphaerella*. In addition, it consequently reduced the abundance of pathogenic fungi and incidence of postharvest decay during the storage of ‘Yali’ pear. The future thrust of this study will focus on the isolation of fungi or bacteria from pear fruit surface and identify their roles in causing fruit decay and changes in fruit quality during storage.

## Data Availability

Data of ITS amplicon sequencing of the samples were submitted to NCBI under the Bioproject ID PRJNA835258.
